# Examining the Effects of the Oral Supplement *Biota orientalis* in the Osteochondral Fragment-Exercise Model of Osteoarthritis in the Horse

**DOI:** 10.3389/fvets.2022.858391

**Published:** 2022-06-01

**Authors:** Kathryn A. Seabaugh, Myra F. Barrett, Sangeeta Rao, C. Wayne McIlwraith, David D. Frisbie

**Affiliations:** ^1^Orthopaedic Research Center, C. Wayne McIlwraith Translational Medicine Institute, Colorado State University, Fort Collins, CO, United States; ^2^Department of Clinical Sciences, Colorado State University, Fort Collins, CO, United States

**Keywords:** horse, osteoarthritis, joint, prostaglandin E (PGE2) 2, oral joint supplement

## Abstract

Osteoarthritis (OA) is a common problem in horses. Several oral supplements have been proposed as treatments for horses with OA. The object of this study was to evaluate the use of the oil extract from the seeds of *Biota orientalis* (BO) for the treatment of experimentally induced OA in horses. OA was induced in 16, 2–5 year old horses in one middle carpal joint on Day 0; the other limb underwent a sham operation. Once daily oral treatment with BO or placebo was initiated on Day 0 and continued to Day 70. All horses were exercised 5 days a week starting on Day 14 through Day 70. The horses were evaluated every other week for lameness and serum GAG concentration as well as weekly synovial fluid assessment. Magnetic resonance imaging was performed on Day 7 and 70. Radiographic changes were assessed on Day 0, 14, and 70. On Day 70 tissue from the middle carpal joint was assessed macroscopically and histologically. All outcome parameters were compared between treatment groups to identify effects of treatment. Compared to placebo a significant decrease was found in synovial fluid prostaglandin E2 concentration and white blood cell counts in horses treated with BO. There was a significant reduction in radiographic scores for subchondral lysis of the radial carpal bone, osteophyte formation, subchondral sclerosis of the radial carpal bone, and total radiographic score for the horses treated with BO. There was no significant difference between treatment groups in clinical lameness findings, MRI findings, macroscopic grading or histologic grading. This study suggests a significant anti-inflammatory effect from oral BO that should be further investigated in clinical OA.

## Introduction

Post-traumatic osteoarthritis (PTOA) is a common cause of discomfort and poor performance in horses (1). It is the most common and economically significant cause of lameness in horses (1, 2). Based upon recent estimations, OA affects almost 3 million horses annually in the United States (1, 3). Continued progression of PTOA can be debilitating, resulting in a decreased quality of life. Treatment in the early stages may help to slow the progression of PTOA and improve the longevity of our equine athlete's careers and quality of life for companion horses. Diagnosis and treatment of PTOA can be timely and expensive. There is no known cure for PTOA and therefore a focus on preventative measures is paramount.

The use of oral supplements in equine medicine is rapidly growing into a global multibillion-dollar industry (4). In a 2017 report, a survey of horse owners found that 30% of respondents spend >$60 per month on supplements for one horse (5). Multiple studies have identified oral joint supplements as one of the most frequently administered supplements to horses (2, 5, 6). Manufacturers of oral joint supplements often included generous claims about their products ability to support the joint leading to stronger articular components which are less prone to degradation. An equine oral joint supplement (Sasha's Blend or 4CYTE^TM^ Equine Granules, Interpath Global Ballarat, Australia) that includes a proprietary oil extract from the seed of *Biota orientalis* (BO); also known as *Thuja orientalis*, has previously been evaluated, resulting in promising *in vivo* and *in vitro* data (7, 8). The oils from the BO seeds have been shown to reduce arachidonic acid release from phosphatidylcholine in the liver of rats (9). Additional *in vitro* data found that BO was able to reduce the production of prostaglandin E_2_ (PGE2) in a porcine cartilage explant model (7). In an acute synovitis model in the equine middle carpal joint, horses that received a dietary nutraceutical, which contained BO, did not exhibit the increases in synovial fluid PGE2 and glycsoaminoglycans (GAG) which typically occurs following synovitis induction (8). Clinically, BO, as a stand-alone ingredient was evaluated in dogs with naturally occurring OA (10). These dogs demonstrated improved objective lameness assessment following 28 days of treatment (10).

The purpose of this study was to test the efficacy of BO as a stand-alone ingredient using a well-established equine model of surgically induced PTOA. Based on the repeatable reduction in PGE2 in previous work (7, 8) as well as improved clinical outcomes in other species (10), it was hypothesized that BO, formulated and marketed as a stand-alone oral joint supplement (4CYTE Epiitalis Forte Gel Horse, Interpath Global Ballarat, Australia), would result in reduced synovial concentrations of PGE2 as well as improved clinical outcomes and evidence of disease modification.

## Materials and Methods

### Horses

Sixteen horses were included in the study. The study was approved by the institution's animal care and use committee (Protocol 18-8072A). The horses were all Quarter Horses or Quarter Horse(s) crosses and a mix of mares and geldings. Pre-study evaluations included a subjective lameness examination, assessment of health and fitness, evaluation for the presence of effusion in middle carpal joints, and radiography of the carpal joints. Horses without abnormal radiographic findings and a subjective lameness score of ≤ 1/5 on a straight line (11) [scale, 0 (normal gait) to 5 (non-weight bearing lameness)], began study acclimatization. Radiographic views for the carpus included lateromedial, dorsopalmar, dorso30°medial palmarolateral oblique (DMPLO), dorso45°lateral palmaromedial oblique (DLPMO) and flexed lateromedial projections. An acclimatization period of 14 days was provided to condition the horses to exercise on a high-speed treadmill prior to data collection.

### Treatments

Eight horses were randomly assigned to the BO-treated [2.5 mL of liquid gel suspension orally, containing 2.2 mL of BO (4CYTE Epiitalis Forte Gel Horse, Interpath Global Ballarat, Australia) and 0.3 mL of excipients] or placebo-treated (2.5 mL of liquid gel suspension orally, containing excipients only) group. Horses were ranked based on their Day 0 lameness examination and then assigned to a treatment group in an alternating manner. The same dose of BO and placebo was administered daily for the duration of the study (10 weeks). Treatments were initiated on Day 0 and administered daily in the evenings for the duration of the study. BO treatments and placebo treatments were placed in color coded dosing syringes and administered directly into the mouth. The code was not revealed to investigators until all data had been collected and analyzed.

### Experimental Induction of Osteoarthritis

On Day 0, horses were anesthetized and routinely prepared for surgery. OA was surgically induced in 1 randomly selected middle carpal joint using a previously described model (12). Briefly, an 8 mm osteochondral fragment was created on the distal aspect of the radial carpal bone at the level of the synovial plica. The fragment was allowed to remain adhered to the joint capsule. The fragment gap was widened to 15 mm using a motorized arthroscopic burr (Arthrex, Munich, Germany). The fragment and bone debris were not removed from the joint as previously described (13). Diagnostic arthroscopy was performed on the contralateral middle carpal joint to confirm the absence of any significant lesions.

Phenylbutazone paste (2g, Vetribute, VetOne, Boise, Idaho, United States) was administered per os once daily for 5 days beginning just prior to experimental induction of osteoarthritis. It was administered in the morning so as not to interfere with absorption of test treatment which was administered in the evening. In accordance with the ACUC protocol, all horses received gentamicin [6.6 mg/kg, intravenous, once (Gentamicin, VetOne, Boise, Idaho, United States)] and cefazolin [11.0 mg/kg IV, twice, 8 h apart (Cefazolin, Hikma Farmaceutica, Portugal)] peri-operatively. The arthroscopic portals were closed with 2-0 nylon suture in a simple interrupted pattern. Both carpi were bandaged in a routine manner. Bandages were changed every 3–5 days for 14 days at which point sutures were removed.

### Exercise Protocol

Horses were housed in individual stalls (3.65 x 3.65 m). A standardized exercise protocol used in this model previously (14) was used to aid in the induction and progression of carpal joint osteoarthritis. Exercise was initiated on day 14 on a high-speed treadmill (EquiGym, Lexington, KY, United States). All horses were trotted for 2 min (4.0–5.0 m/s), galloped for 2 min (9.0–10.0 m/s) and trotted again for 2 min (4.0–5.0 m/s) 5 days a week for the duration of the study starting on Day 14.

### Lameness

Clinical examination and lameness examinations for both thoracic limbs were performed by a board-certified equine sports medicine specialist (KAS) blinded to the treatment group at day −7 (baseline), day 14 and every other week thereafter throughout the study period. All horses had been acclimated to the treadmill prior to acquiring the baseline lameness data so as to minimize any effect of the treadmill on lameness. Subjective lameness grading used the American Association of Equine Practitioners' lameness scale (11) [scale, 0 (normal gait) to 5 (non-weight bearing lameness)]. Simultaneously, objective lameness data, specifically fore signed vector sum, was collected using an inertial sensor system (Equinosis® Lameness Locator, Columbia, Missouri, United States) (15). The fore signed vector sum indicates the direction and magnitude of lameness; positive values indicating a horse is right front lame and a negative value indicating a horse is left from lame. The vector represents the magnitude as millimeters of displacement. Clinical lameness represents anything greater or less than 8.5 mm of displacement. At each lameness examination the horses were also assessed for carpal effusion and response to flexion using a subjective ordinal grading scale of 0 to 4 (0 = normal, 1= slight, 2 = mild, 3 = moderate, 4 = severe/marked).

### Diagnostic Imaging

Radiographic evaluations of both carpi were performed prior to inclusion into the study (baseline), day 14, and 70 utilizing 5 standard views (lateromedial, dorsopalmar, dorso30°medial palmarolateral oblique (DMPLO), dorso45°lateral palmaromedial oblique (DLPMO) and flexed lateromedial). A board-certified veterinary radiologist (MBF) who was unaware of treatment group assignment graded the carpal radiographs for osseous proliferation at the dorsal joint capsule attachment of the radial carpal bone (enthesopathy), subchondral bone lysis of the radial carpal bone, subchondral sclerosis of the radial carpal bone, subchondral sclerosis of the third carpal bone and osteophyte formation. A previously established ordinal grading scale (14) of 0 to 4 was used for each radiographic outcome parameter (0 = no detectable abnormality, 1 = slight change, 2 = mild change, 3 = moderate change, and 4 = severe change). A total radiographic score was also calculated for each limb based on a summation of the scores from the 5 radiographic outcome variables (Total Rad Score).

Magnetic resonance imaging (MRI) was performed under general anesthesia at Day 7 and after euthanasia at Day 70 of bilateral carpi. A 1.0 tesla extremity scanner (ONI, GE, Boston, MA, United States) was utilized for the MRI examinations. Fast spin echo (FSE), proton density, short tau inversion recovery (STIR), T1 and T2 weighted images were obtained in sagittal, transverse and dorsal planes. The MRI examinations were subjectively graded by a board-certified veterinary radiologist (MBF) with extensive experience who was blinded to the treatment groups for degree of synovial fluid, synovial proliferation, joint capsule thickness, joint capsule edema, joint capsule fibrosis, radial carpal bone edema-like signal, radial carpal bone sclerosis, and third carpal bone sclerosis. A subjective ordinal grading scale of 0 to 4 was used for each MRI outcome parameter (0 = no detectable abnormality, 1 = slight change, 2 = mild change, 3 = moderate change, and 4 = severe change). This grading system is modified from a previously reported carpal study (16) and closely represents scoring utilized in a recent osteochondral study in the stifle of the horse (17). A total MRI score was also calculated for each limb based on a summation of the scores for the 8 MRI outcome variables.

### Synovial Fluid

Synovial fluid (SF) was collected from both middle carpal joints weekly throughout the study except for week 1 (day 7). Synovial fluid collected at endpoint was collected following the post-mortem MRI examinations. The synovial fluid collected from each timepoint was equally divided into an ethylenediamine tetraacetic acid (EDTA) blood tube and serum blood tube. The synovial fluid in the EDTA blood tube was analyzed for cellularity and protein concentrations through a clinical pathology laboratory. The fluid was refrigerated and analyzed within 12 h. The synovial fluid placed in the red top tube was spun down and the supernatant was placed in plastic microtubes and frozen at−80°C until it was analyzed for PGE2 and glycosaminoglycan (GAG) concentrations as previously described (18, 19).

For SF PGE2 concentrations, the SF samples were first subjected to solid-phase extraction procedures per manufacturers protocol (Bond Elut C-2 mini columns, Agilent Technologies, Santa Clara, CA, United States) to extract the PGE2. The dried, extracted PGE2 samples were stored at 4°C or immediately used. The PGE2 was then quantified utilizing a commercially available enzyme immunoassay (PGE2 ELISA kit, Enzo Life Sciences, Inc., Farmingdale, NY, United States) using a monoclonal antibody to bind to the PGE2. The dried PGE2 was rehydrated with the supplied assay buffer. The rehydrated sample was placed in triplicate wells, coated with a goat antibody specific to mouse IgG and incubated on a shaker for 2 h. The plate was then washed and the manufacturers substrate was added and the wells were incubated for 45 min and then stop solution was applied. Absorbance of each well was measured using a multimode microplate reader (Spectramax M3, Molecular Devices, San Jose, CA, United States). PGE2 concentrations were extrapolated using the established standard curve.

For SF GAG concentrations, 15 uL of synovial fluid was placed in the wells in triplicate with 185 uL dimethylmethylene blue (DMMB). The plate was read within 10 min of mixing the sample and dye with a multimode microplate reader. GAG concentrations were extrapolated using the established standard curve. Standards were compared to chondroitin sulfate C from shark cartilage.

### Serum

Blood was collected from the jugular vein on days 0, 14, 28, 42, and 70. The blood was collected into a red top tube and allowed to sit for 30 min before centrifugation. The serum was harvested and stored in plastic microtubes at−80°C until analysis. The serum was used to measure GAG concentrations using the same process as described above for synovial fluid.

### Post-mortem Examination

Horses were euthanized at the completion of the study (Day 70) with an overdose of sodium pentobarbital sodium (Euthanasia Solution, VetOne, Boise, ID, United States) given IV. Immediately following euthanasia, both carpi underwent MRI as described above. At the completion of the MRI, synovial fluid was collected from each middle carpal joint. The middle carpal joints were then disarticulated and the joints were graded immediately (KAS) using a subjective ordinal scale (12) for total erosion, total hemorrhage, full thickness and partial thickness erosions [0 (normal) to 4 (severe)]. The presence of an osteochondral fragment, synovial adhesion, and kissing lesion were noted (yes or no) but not graded. The person grading the joints (KAS) was not blinded to the presence or absence of a fragment, they were however blinded to treatment group assignments.

**Figure 1:**
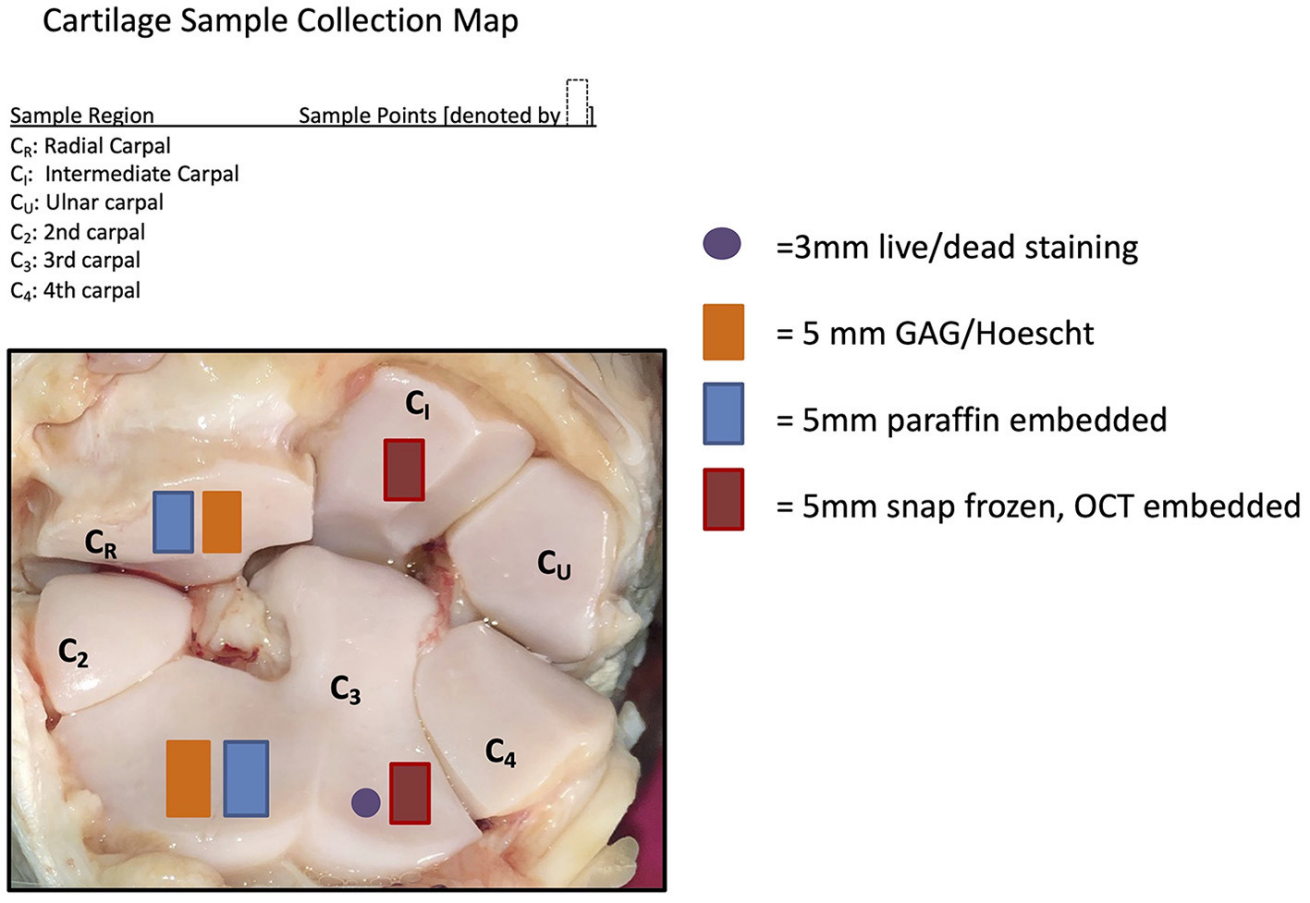
Cartilage sample collection map demonstrating where the samples were collected for the post-mortem sampling.

Samples of synovial membrane and articular cartilage were harvested during necropsy following macroscopic examination. The samples were placed in neutral-buffered 10% formalin and processed for histologic examination. Cartilage and synovium samples were sectioned and stained by Hematoxylin and Eosin (H&E) to determine cellular changes. Cartilage samples were stained by Safranin O and Fast Green (SOFG) to subjectively assess changes in GAG content. Cartilage sample locations are demonstrated in Figure 1. Histology was graded by a single evaluator (DDF) using a modified Mankin scoring system (20).

Cartilage samples collected from the radial carpal bone and third carpal bone (Figure 1) were used for quantification of total GAG concentrations using a dimethylmethylene blue (DMMB) assay as previously described (12, 21), as well as analysis for cell viability and apoptosis.

Cell viability was assessed using a commercially available kit (Live/Dead Viability/Cytotoxicity Kit, L3224, Invitrogen Inc, Carlsbad, CA, United States) for mammalian cells. The kit provides a two-color fluorescence cell viability assessment. When calcein AM and ethidium homodimer are added to the cartilage, the live cells, containing intracellular esterases, cleave the non-fluorescent, cell-permeant calcein AM molecules to calcein. The calcein is retained in live cells and produces intensely green fluorescence. Specialized software (CellSens Dimension Software, Olympus Corporation, Center Valley, PA, United States) was utilized to capture images and perform automated counts of both stained cell populations. Cell counts were normalized to tissue mass.

Apoptotic status of cartilage samples was assessed using an apoptosis kit (Millipore Sigma Apoptag© Peroxidase *in Situ* Apoptosis Detection Kit, S7100; Burlington, MA, United States). The kit distinguishes apoptosis from necrosis by detecting DNA cleavage and chromatin condensation, both hallmark characteristics of apoptosis. The slides were prepared per manufacturer protocol. Apoptotic cells were calculated by manual counting by two individuals and averaged.

### Statistical Analysis

As OA and sham surgeries were conducted on each horse, statistical analyses were conducted on the difference of the variable values measured at each surgical site to reduce the total level of variation in the data. Furthermore, the baseline values were subtracted from the observations from later time periods, to investigate how the variables of interest changed during the study. That is, the values used in the analyses for horse *i* at time *t* (*d*_*i, t*_) were calculated as:


$$\begin{array}{l} d_{i,t}=\left(x_{i,OA,t}-x_{i,sham,t}\right)-\left(x_{i,OA,base}-x_{i,sham,base}\right) \end{array}$$

Therefore, all results are in terms of changes from baseline. Linear mixed-models were used for analyses where repeated measurements from the same horses were included in the analyses (e.g., where bi-weekly measurements were used), with horse ID used as a random effect on the intercept term. The predictor variables *week* and *treatment group* (placebo or BO) were included as fixed, additive, effects. Linear models were used where only the change at endpoint data was considered for analysis, with *treatment group* specified as a fixed effect. When only one observation per horse was recorded (i.e., serum GAG concentrations), a linear model was used to analyze the data. Baseline data was again subtracted from the observations resulting in analyses of the difference from baseline. Objective lameness data collected via inertial sensors reported one observation per horse for forelimb lameness (i.e., the lameness scores was only assigned to either the left front or the right front); therefore, assessments were performed as a change from baseline. Chi-square or *t*-tests were used to determine the significance of the *treatment group* effects, with *p*-values <0.05 considered statistically significant. No adjustments were made for testing multiple variables. Diagnostic checks did not indicate any concerning violations of model assumptions.

Statistical analyses were performed on the difference from baseline as described above. Descriptive statistics were also calculated for all variables and are reported as mean +/- SEM (95% CI).

## Results

### Horses

Sixteen Quarter Horses or Quarter Horse crosses were included in the study. The mean age was 3 years old (range 2–5 years old) based on dental age. Six mares and 10 geldings were included in the study. The mean weight for all horses was 379 kg (range 329–452 kg). Horses were randomly assigned to groups which resulted in 2 mares and 6 geldings in the treatment group and 4 mares and 4 geldings in the placebo group.

### Clinical Examination

**Figure 2:**
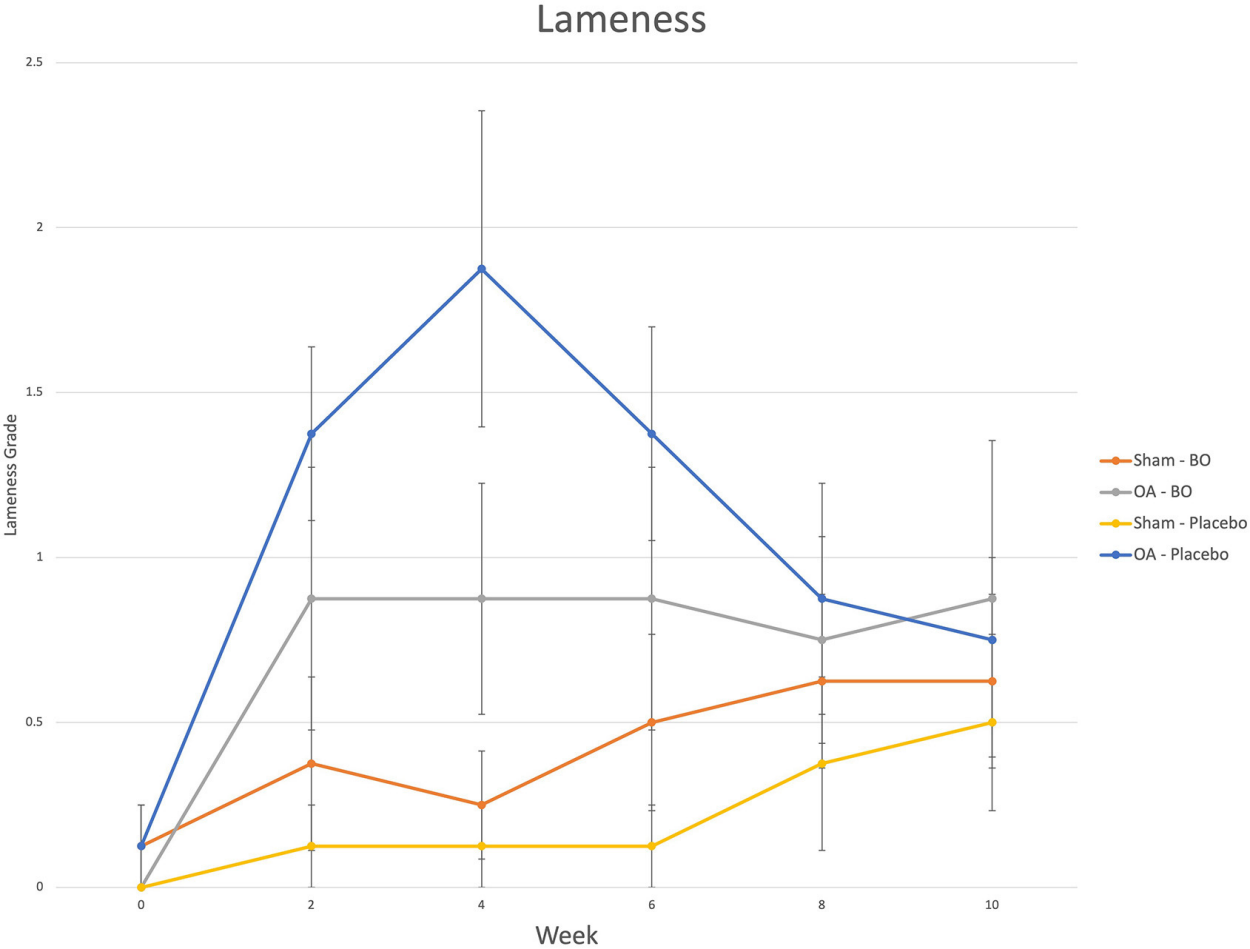
Subjective lameness grades (0–5) were assigned to each limb on weeks 0, 2, 4, 6, 8 and 10. The means are represented for the OA limb of horses receiving the treatment (OA-BO), sham operated limb of horses receiving the treatment (Sham-BO), OA limb of horses receiving the control (OA-Placebo) and sham operated limb of horses receiving the control (Sham-Placebo). This data represents mean (+/- SEM) from raw data and not the data upon which the statistics were run thereby a *p*-value does not correlate with the data presented here.

**Figure 3:**
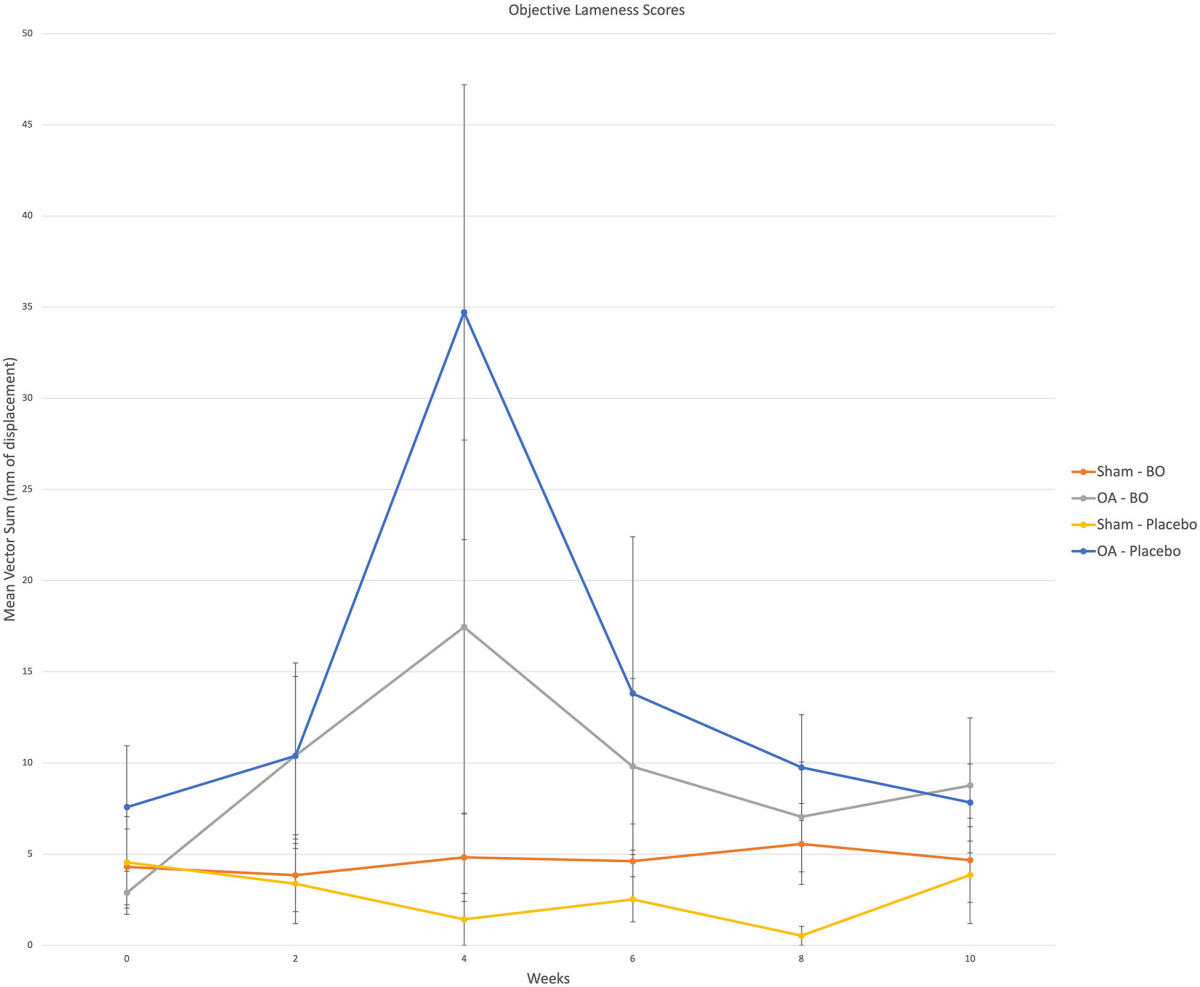
Objective lameness scores were obtained via an inertial sensor system on weeks 0, 2, 4, 6, 8 and 10. A single fore signed vector sum was assigned to each horse for which a positive value represented a right front lameness and a negative value represented a left front lameness. The value represents a magnitude of lameness in the form of millimeters of displacement. For analysis the (+) and (-) were removed as the values were assigned to the specific limb; i.e., OA limb of horses receiving the treatment (OA-BO), sham operated limb of horses receiving the treatment (Sham-BO), OA limb of horses receiving the control (OA-Placebo) and sham operated limb of horses receiving the control (Sham-Placebo). This data represents mean (+/- SEM) from raw data and not the data upon which the statistics were run thereby a *p*-value does not correlate with the data presented here.

There were consistent clinical effects observed with visual evaluation in all horses by day 14 as a result of the OA model; joints with OA induction demonstrated greater effusion scores, flexion scores and lameness scores than the sham operated joints. Over the course of the study, there was no significant effect of time or treatment on the clinical measurements of middle carpal joint effusion, subjective lameness grades nor response to carpal flexion. Mean subjective lameness scores from the raw data are represented in Figure 2. There were no statistically significant differences identified in any time point (*p* = 0.41) for the objective lameness scores. The mean objective lameness data from the raw data is represented in Figure 3.

### Synovial Fluid Analysis

**Figure 4:**
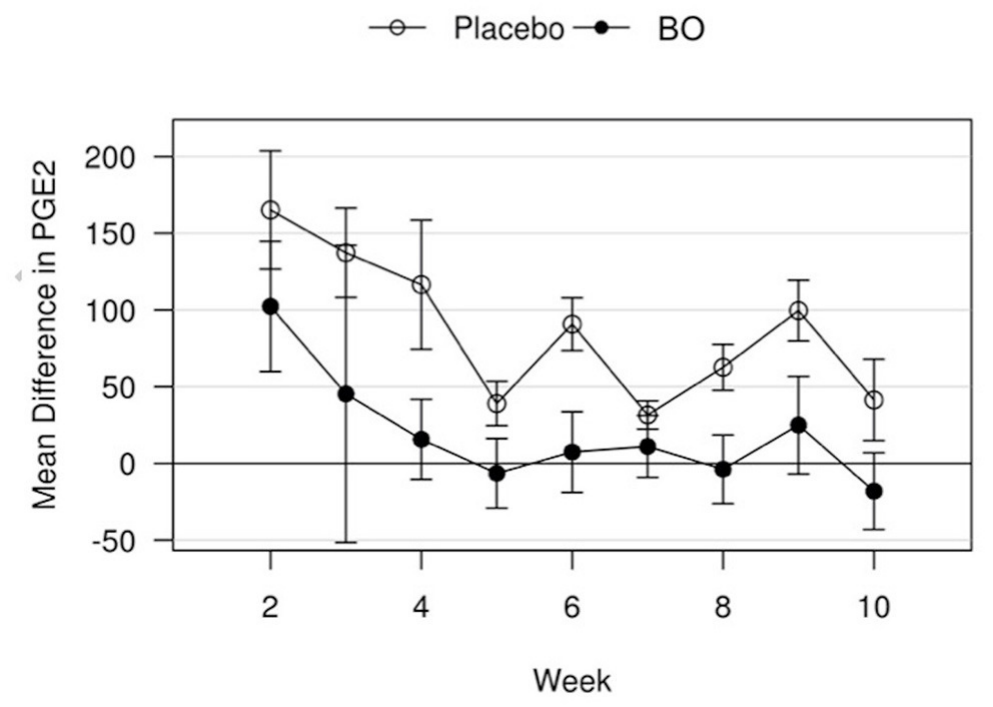
Mean differences in PGE2 values for placebo and BO treated horses. Measurements are in terms of changes from baseline. Values were also adjusted to accommodate the values of the sham operated joint, such that the means represented here are based on the following equation: *d*_*i, t*_ = (*x*_*i, OA, t*_−*x*_*i, sham, t*_)−(*x*_*i, OA, base*_−*x*_*i, sham, base*_). A significant difference (p = 0.043) was found when evaluating across all time points, but no single time point was significantly different. Error bars represent +/- 1 standard error. Units are pg/mL.

**Figure 5:**
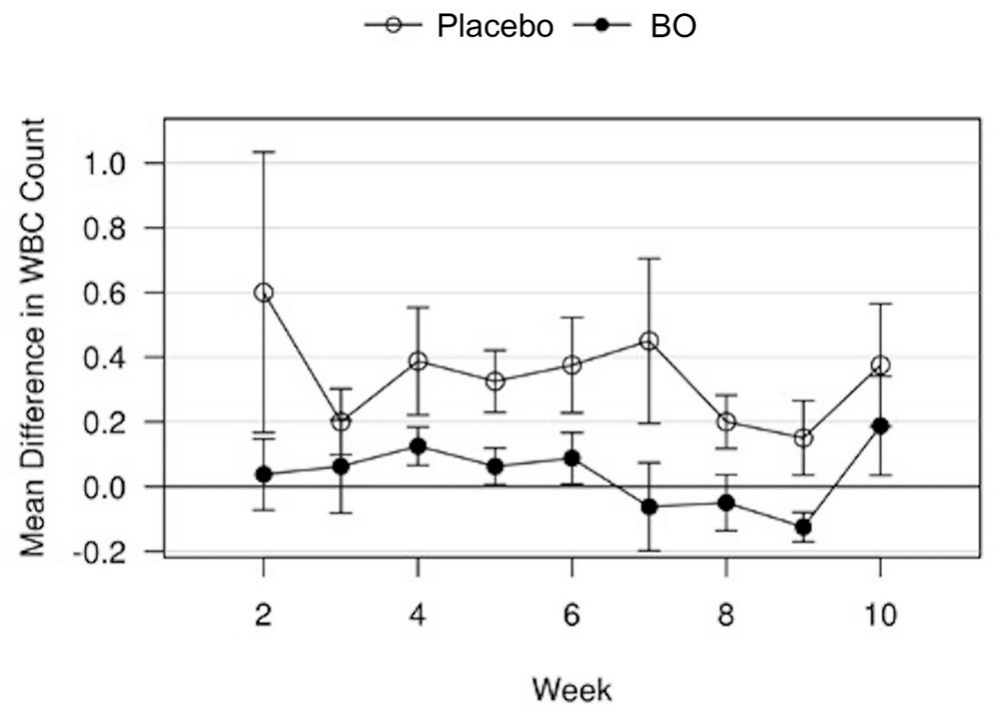
Mean differences in synovial white blood cell (WBC) counts for placebo and BO treated horses. Measurements are in terms of changes from baseline. Values were also adjusted to accommodate the values of the sham operated joint, such that the means represented here are based on the following equation: *d*_*i, t*_ = (*x*_*i, OA, t*_−*x*_*i, sham, t*_)−(*x*_*i, OA, base*_−*x*_*i, sham, base*_). A significant difference (*p* = 0.005) was found when evaluating across all time points, but no single time point was significantly different. Error bars represent +/- 1 standard error. Units are cells x 10^3^/uL.

A median volume of 3.0 mL of synovial fluid (range 1.5–5.5 mL) was collected at each timepoint. A significant treatment effect (*p* = 0.043) was found with lower PGE2 concentrations found in synovial fluid of horses receiving BO compared to the placebo (Figure 4), when analyzing the differences from baseline and assuming a constant difference between groups. From the random effects model the difference was estimated to be−67.29 pg/mL [SE = 31.17, 95%CI = (–133.37, −1.21)]. A significant treatment effect (*p* = 0.005) was found with lower white blood cell counts found in synovial fluid of horses receiving BO compared to placebo (Figure 5), when analyzing the differences from baseline and assuming a constant difference between groups. From the random effect model the difference was estimated to be −0.3042 cells x 10^3^/uL [SE = 0.0950, 95%CI = (–0.5056, −0.1027)]. The raw data (mean +/- SEM) for synovial fluid total protein, PGE2 and white blood cell counts have been included in Supplementary Table 1. There was no significant treatment effect on GAG concentrations in synovial fluid. There was no significant treatment effect upon synovial fluid total protein.

### Serum Analysis

There was no significant treatment effect on total serum GAG concentrations.

### Diagnostic Imaging

**Figure 6:**
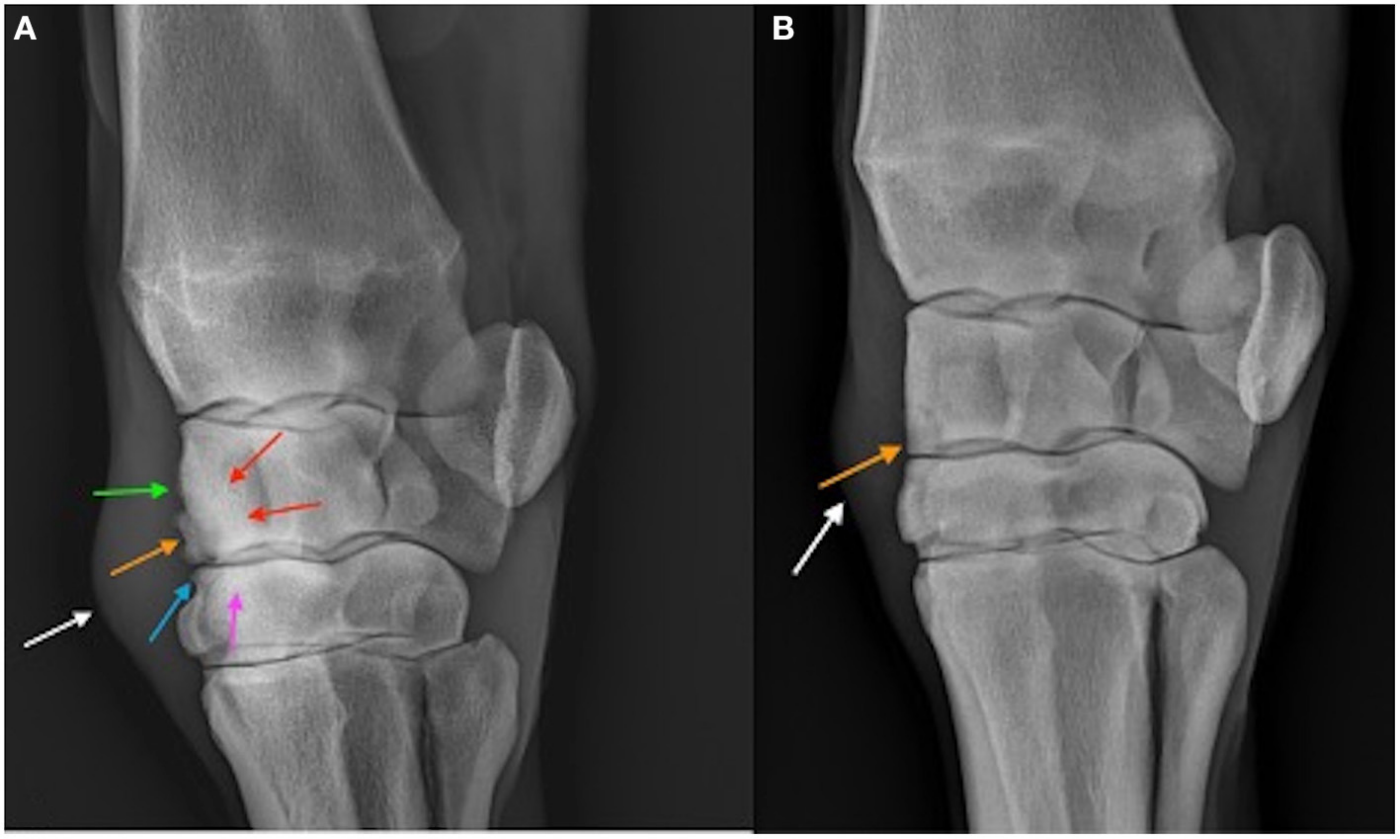
Dorsolatero-palmaromedial oblique radiographs of a placebo treated horse **(A)** and BO treated horse **(B)** on Day 70. The osteochondral fragment is visible in both horses (orange arrow). Sclerosis of the third carpal bone (pink arrow) and radiocarpal bone (red arrows) are visible in the placebo treated horse as well as enthesopathy of the joint capsule (green arrow), and osteophytosis (blue arrow). Soft tissue swelling (white arrow) representing middle carpal joint effusion is the only common abnormality between the two horses.

When comparing the difference from baseline at 2 and 10 week there were significantly lower radiographic scores in the treated horses for subchondral bone lysis of the radial carpal bone (*p* = 0.019), osteophyte formation (*p* <0.001), subchondral sclerosis of the radial carpal bone (*p* = 0.012), and total radiographic score (*p* = 0.001) for the horses treated with BO (Figure 6). The means and estimates of variance are represented in Table 1. There was no significant treatment effect on enthesopathy nor subchondral sclerosis of the third carpal bone although there was trend toward significance for the latter (*p* = 0.054). The raw radiographic scores (mean +/- SEM) have been included in Supplementary Table 2.

**Table 1:** Means and estimates of variance for radiographic parameters that demonstrated a significant difference between the BO-treated and placebo-treated horses when evaluating a difference from baseline.

		**Estimated difference between groups**	**SEM**	**Lower 95% C.I**.	**Upper 95% C.I**.
Subchondral bone lysis of RC bone	Week 2	0.000	0.269	−0.570	0.570
	Week 10	−0.750	0.269	−1.320	−0.180
Osteophyte formation	Week 2	0.000	0.185	−0.392	0.392
	Week 10	−1.125	0.185	−1.517	−0.733
Subchondral sclerosis of RC bone	Week 2	−0.250	0.352	−0.997	0.497
	Week 10	−1.125	0.352	−1.872	−0.378
Total radiographic score	Week 2	−0.250	0.989	−2.347	1.847
	Week 10	−4.000	0.989	−6.097	−1.903

There was no significant treatment effect on the MRI scores.

### Postmortem Examination

There was no significant effect of treatment on macroscopic grading for total cartilage erosion, total synovial hemorrhage, nor full thickness or partial thickness cartilage erosions.

There was no significant effect of treatment on histologic evaluation of synovial membrane nor articular cartilage.

There was no significant effect of treatment on GAG concentrations in articular cartilage. There was no significant effect of treatment on the percent of live cells in the cartilage ascertained from the cell viability staining. Nor was there a significant treatment effect upon the number of apoptotic cells within the cartilage.

## Discussion

In this in vivo model of osteoarthritis significant anti-inflammatory and disease-modifying effects were seen following oral treatment with BO. The most relevant findings pertained to the concentration of reduced concentration of PGE2 in synovial fluid and the reduction of radiographic signs of osteoarthritis compared to horses in the placebo group.

The concentration of PGE2 in synovial fluid is commonly evaluated as a measurement of inflammation within a joint and is consistently increased in this experimental OA model (22). Significantly greater synovial fluid PGE2 concentrations have been found between horses with OA undergoing exercise than those horses undergoing exercise alone (23). In addition, increased synovial fluid PGE2 has been reported in horses with naturally occurring OA (24), as well as lameness in horses (25) and dogs (26). The reduction of PGE2 concentration in the current study suggest a significant anti-inflammatory effect from BO administered orally.

White blood cell counts within synovial fluid are commonly utilized as an indicator of inflammation. In equine synovial fluid, a white blood cell count <1.0 x 10^3^ cells/uL is considered normal (27). With the exception of Day 70 (OA joint in the placebo group = 1.20 x 10^3^ cells/uL), the mean white blood cell count did not exceed 1.0 x 10^3^ cells/uL. That withstanding, white blood cell counts were significantly lower in the BO treated horses. The lower white blood cell counts in BO treated horses may correlate with an additional anti-inflammatory effect of the BO treatment.

Radiographic changes are known to lag behind other clinical symptoms of OA. In this model of OA, subtle changes are common. Due to this, radiographs are often an outcome parameter with low sensitivity for disease modification but considered by the investigators to be highly specific. In the current study, significant reduction in radiographic changes with treatment was seen in 4 of the 6 radiographic parameters evaluated. At endpoint, the total radiographic score of BO treated horses was 63% lower than placebo treated horses (2.38 vs. 6.37, respectively). These improvements in radiographic outcome parameters are some of the most compelling seen in numerous iterations of this model and represent a noteworthy benefit of this treatment in the authors' opinion. This finding is strongly supportive of a disease modifying effect with BO treatment.

MRI is recognized as a highly valuable imaging modality. The authors feel that there are three potential explanations for a lack of significance in MRI findings. First, the timing of the baseline MRI examination was at day 7, after OA induction. Though this timeline was chosen to allow comparison of the osteochondral fragment over time, it did not provide a true baseline MRI examination. By day 7, there were already changes associated with nearly all the MRI criteria. The acute nature of the chip on Day 7 can result in artifacts which can inhibit true assessment of the MRI changes. Second, it is known that due to volume averaging and signal characteristics, small osteophytes or enthesophytes may be occult on MRI but visible on radiographs. Third, the high sensitivity of MRI may have detected subtle changes in subchondral bone lysis making the difference between the presence or absence of bone changes less significant. In conclusion, the greater detail available with MRI is valuable but can result in the need for a greater sample size to detect a significant difference.

The timing of the MRI examination in this study represents a limitation for detection of a significant difference in treated animals compared to controls. In this study the MRI examinations were performed at Day 7 and 70. While performing the MRI examination on Day 7 allowed for assessment of the osteochondral fragment over time (Day 7 and 70) it neglects the degree of OA that occurred between Day 0 and 7, which is known to be substantial. Specifically, this model has demonstrated evidence of OA in the first 14 days that was not dissimilar to that observed in other examples of this model at Day 70 (28). In the current study, all the OA limbs, had at least one MRI parameter that was graded as mild change (grade 2) by Day 7, suggesting progression of OA at this early timepoint. Thus, the lack of significant difference in MRI parameters may have been in part due to the degree of OA already present at Day 7, the first MRI examination point.

An additional limitation is the initiation of treatment on Day 0. This did not allow for grouping of horses based on their lameness scores following OA induction, nor could we make clear distinctions between baseline, OA induction and treatment initiation. The initiation of treatment on Day 0 has been performed before however using this model (22) and was chosen based on the expected prophylactic clinical treatment usage of this product.

It should be stated that an additional limitation to the study is that a single (but separate) reviewer was used for the grading of the clinical examination, gross examination, the imaging (MRI and radiography) and the histology. This model is well established, and the reviewers all have extensive experience utilizing the specified grading scales. It should be acknowledged however that there has been no assessment of the repeatability and reproducibility of these assessments. It is of the author's opinion that due to the multiple studies each of the reviewers have participated in as well as their clinical experience that the intra-observer repeatability would be high.

This proprietary extract from *Biota orientalis* has been evaluated in various models (7–10, 29) and similar beneficial outcomes have been observed lending enhanced credibility to the findings of the current study. BO is a highly bioavailable fatty acid. The mechanism by which fatty acids can influence inflammation are varied and the mechanism by which BO influence PGE2 production is unknown. Previous research would suggest it may be due to the effect on arachidonic acid release (9) as PGE2 is released via oxidation of arachidonic acid (7).

In this study, the authors hypothesized that BO, as a stand-alone ingredient, would reduce synovial PGE2 concentrations resulting in anti-inflammatory effects. This hypothesis was confirmed with a significant decrease in synovial PGE2 and white blood cell concentrations following oral treatment with BO in this equine *in vivo* model of OA. BO, a proprietary oil extract, has shown similar results in other studies displaying a consistent anti-inflammatory effect. The disease modifying effects, as demonstrated by the reduction in radiographic changes, of this treatment compared to other therapeutics evaluated using this model suggest a substantial potency that is greater than other parenteral therapeutic and oral products. These results in combination with the previous *in vitro* studies looking at the same product provides strong support for its use as a prophylactic therapy for slowing the production of OA in horses.

## Data Availability Statement

The raw data supporting the conclusions of this article will be made available by the authors, without undue reservation.

## Ethics Statement

The animal study was reviewed and approved by Colorado State University Animal Care and Use Committee Protocol 18-8072A.

## Author Contributions

KS was critical to study design, data collection, and manuscript preparation. DF and CM also assisted with study design, data collection, and manuscript preparation. SR assisted with data analysis and manuscript preparation. MB reviewed the imaging and assisted with manuscript preparation. All authors contributed to the article and approved the submitted version.

## Funding

This work was supported by Interpath Global, Ballarat, Australia. All authors listed have no personal financial interest or conflict of interest with the stated company.

## Conflict of Interest

The authors declare that the research was conducted in the absence of any commercial or financial relationships that could be construed as a potential conflict of interest.

## Publisher's Note

All claims expressed in this article are solely those of the authors and do not necessarily represent those of their affiliated organizations, or those of the publisher, the editors and the reviewers. Any product that may be evaluated in this article, or claim that may be made by its manufacturer, is not guaranteed or endorsed by the publisher.
