# Supplementary material for: Examining the Effects of the Oral Supplement Biota orientalis in the Osteochondral Fragment-Exercise Model of Osteoarthritis in the Horse
Source: Front Vet Sci. 2022 Jun 1;9:858391. doi: 10.3389/fvets.2022.858391 (PMC9198577; doi:10.3389/fvets.2022.858391)
Supplement: Supplementary file 1 [file Data_Sheet_1.docx]

| Day | Treatment | Joint | SF Total Protein  (g/dL) | | PGE2  (pg/mL) | | White Blood Cell Count (cells x 10^3^/uL) | |
| --- | --- | --- | --- | --- | --- | --- | --- | --- |
|  |  |  | Mean | SEM  (95% CI) | Mean | SEM  (95% CI) | Mean | SEM  (95% CI) |
| 0 | BO | Sham Joint | 2.14 | 0.21  (1.64-2.63) | 70.41 | 9.31  (48.40-92.42) | 0.28 | 0.04  (0.19-0.36) |
|  |  | OA Joint | 2.13 | 0.18  (1.69-2.58) | 98.32 | 17.85  (56.12-140.53) | 0.39 | 0.05  (0.26-0.52) |
|  | Placebo | Sham Joint | 1.93 | 0.12  1.64-2.21) | 101.35 | 15.22  (65.36-137.35) | 0.38 | 0.07  (0.22-0.53) |
|  |  | OA Joint | 1.85 | 0.18  (1.41-2.29) | 96.23 | 15.93  (58.55-133.91) | 0.31 | 0.05  (0.19-0.43) |
| 14 | BO | Sham Joint | 1.23 | 0.11  (0.96-1.49) | 68.12 | 7.15  (51.22-85.03) | 0.23 | 0.02  (0.19-0.26) |
|  |  | OA Joint | 2.35 | 0.18  (1.93-2.78) | 198.36 | 34.04  (117.88-278.85) | 0.38 | 0.08  (0.18-0.57) |
|  | Placebo | Sham Joint | 1.25 | 0.17  (0.84-1.66) | 80.11 | 9.98  (56.51-103.71) | 0.26 | 0.04  (0.17-0.35) |
|  |  | OA Joint | 2.55 | 0.24  (1.99-3.11) | 240.15 | 37.05  (152.55-327.75) | 0.80 | 0.42  (-0.18-1.78) |
| 21 | BO | Sham Joint | 1.55 | 0.23  (1.02-2.08) | 114.05 | 34.58  (32.28-195.82) | 0.35 | 0.05  (0.24-0.46) |
|  |  | OA Joint | 2.23 | 0.21  (1.72-2.73) | 187.32 | 53.76  (60.21-314.44) | 0.53 | 0.12  (0.25-0.80) |
|  | Placebo | Sham Joint | 1.33 | 0.13  (1.01-1.64) | 69.22 | 2.29  (63.81-74.62) | 0.29 | 0.04  (0.20-0.37) |
|  |  | OA Joint | 2.66 | 0.16  (2.29-3.04) | 201.40 | 36.51  (115.06-287.74) | 0.43 | 0.07  (0.26-0.59) |
| 28 | BO | Sham Joint | 1.35 | 0.14  (1.02-1.68) | 36.99 | 2.81  (30.34-43.64) | 0.23 | 0.03  (0.17-0.28) |
|  |  | OA Joint | 2.21 | 0.24  (1.65-2.77) | 80.58 | 10.77  (55.11-106.05) | 0.46 | 0.05  (0.34-0.58) |
|  | Placebo | Sham Joint | 1.33 | 0.12  (1.05-1.60) | 34.62 | 2.66  (28.33-40.91) | 0.23 | 0.02  (0.19-0.26) |
|  |  | OA Joint | 2.33 | 0.14  (2.00-2.65) | 146.06 | 40.92  (49.29-242-83) | 0.55 | 0.16  (0.17-0.93) |
| 35 | BO | Sham Joint | 1.50 | 0.17  (1.10-1.90) | 70.80 | 6.48  (55.47-86.13) | 0.18 | 0.03  (0.12-0.23) |
|  |  | OA Joint | 2.08 | 0.20  (1.61-2.54) | 92.31 | 13.16  (61.19-123.43) | 0.35 | 0.05  (0.22-0.48) |
|  | Placebo | Sham Joint | 1.45 | 0.09  (1.24-1.66) | 69.98 | 5.31  (57.43-82.53) | 0.19 | 0.02  (0.13-0.24) |
|  |  | OA Joint | 2.00 | 0.11  (1.73-2.27) | 103.93 | 7.83  (85.41-122.45) | 0.45 | 0.07  (0.29-0.61) |
| 42 | BO | Sham Joint | 1.40 | 0.14  (1.07-1.73) | 70.39 | 4.69  (59.29-81.49) | 0.24 | 0.05  (0.12-0.36) |
|  |  | OA Joint | 1.80 | 0.16  (1.43-2.17) | 105.75 | 10.53  (80.86-130.64) | 0.44 | 0.06  (0.30-0.58) |
|  | Placebo | Sham Joint | 1.48 | 0.11  (1.22-1.73) | 77.65 | 6.31  (62.73-92.57) | 0.26 | 0.05  (0.15-0.37) |
|  |  | OA Joint | 1.98 | 0.09  (1.76-2.19) | 163.33 | 21.71  (112.00-214-67) | 0.58 | 0.12  (0.29-0.86) |
| 49 | BO | Sham Joint | 1.53 | 0.10  (1.28-1.77) | 53.078 | 3.80  (44.10-62.06) | 0.38 | 0.09  (0.17-0.58) |
|  |  | OA Joint | 1.78 | 0.16  (1.39-2.16) | 92.02 | 8.91 (70.94-113.09) | 0.43 | 0.05  (0.32-0.53) |
|  | Placebo | Sham Joint | 1.63 | 0.20  (1.16-2.09) | 65.79 | 5.07  (53.79-77.78) | 0.30 | 0.03  (0.24-0.36) |
|  |  | OA Joint | 1.93 | 0.16  (1.54-2.31) | 92.30 | 11.67  (64.70-119.90) | 0.69 | 0.20  (0.21-1.16) |
| 56 | BO | Sham Joint | 1.58 | 0.12  (1.29-1.86) | 83.86 | 11.12  (57.56-110.16) | 0.44 | 0.06  (0.30-0.57) |
|  |  | OA Joint | 1.78 | 0.13  (1.47-2.08) | 107.99 | 19.78  (61.22-154.77) | 0.50 | 0.11  0.23-0.77) |
|  | Placebo | Sham Joint | 1.60 | 0.20  (1.14-2.06) | 77.48 | 9.68  (54.59-100.38) | 0.33 | 0.05  (0.22-0.43) |
|  |  | OA Joint | 1.88 | 0.12  (1.60-2.15) | 135.02 | 17.11  (94.56-175.48) | 0.46 | 0.05  (0.34-0.58) |
| 63 | BO | Sham Joint | 1.58 | 0.15  (1.22-1.93) | 60.72 | 8.73  (40.09-81.36) | 0.33 | 0.04  (0.24-0.41) |
|  |  | OA Joint | 1.73 | 0.18  (1.30-2.15) | 113.64 | 13.06  (82.77-144.51) | 0.31 | 0.04  (0.22-0.41) |
|  | Placebo | Sham Joint | 1.70 | 0.18  (1.28-2.12) | 81.59 | 12.86  (51.18-112.00) | 0.41 | 0.06  (0.28-0.55) |
|  |  | OA Joint | 1.90 | 0.16  (1.52-2.28) | 176.10 | 26.02  (114.56-237.63) | 0.50 | 0.08  (0.30-0.70) |
| 70 | BO | Sham Joint | 1.73 | 0.21  (1.22-2.23) | 73.91 | 9.25  (52.04-95.77) | 0.65 | 0.10  (0.41-0.89) |
|  |  | OA Joint | 2.10 | 0.14  (1.77-2.43) | 83.81 | 11.06  (57.66-109.96) | 0.95 | 0.11  (0.70-1.20) |
|  | Placebo | Sham Joint | 1.78 | 0.08  (1.58-1.97) | 76.42 | 12.35  (47.22-105.63) | 0.89 | 0.15  (0.54-1.23) |
|  |  | OA Joint | 2.05 | 0.10  (1.82-2.28) | 112.72 | 20.46  (64.33-161.10) | 1.20 | 0.14  (0.88-1.52) |

**Supplemental Table 1:** Mean and SEM values for synovial fluid total protein, PGE2 concentrations and white blood cell counts. These values represent raw data and differ from the values that were statistically analyzed therefore no data point correlates with a specific p-value.

| Day | Treatment | Joint | Joint capsule enthesopathy | | Subchondral bone lysis | | Osteophyte formation | | Subchondral bone sclerosis (3^rd^) | | Subchondral bone sclerosis (CR) | | Total radiographic score | |  |
| --- | --- | --- | --- | --- | --- | --- | --- | --- | --- | --- | --- | --- | --- | --- | --- |
|  |  |  | Mean | SEM (95% CI) | Mean | SEM (95% CI) | Mean | SEM (95% CI) | Mean | SEM (95% CI) | Mean | SEM (95% CI) | Mean | SEM (95% CI) |  |
| 0 | BO | Sham Joint | 0 | 0 | 0 | 0 | 0 | 0 | 0 | 0 | 0.13 | 0.13 (-0.17-0.42) | 0.13 | 0.13 (-0.17-0.42) | |
|  |  | OA Joint | 0 | 0 | 0 | 0 | 0 | 0 | 0 | 0 | 0.13 | 0.13 (-0.17-0.42) | 0.13 | 0.13 (-0.17-0.42) | |
|  | Placebo | Sham Joint | 0 | 0 | 0 | 0 | 0 | 0 | 0 | 0 | 0.25 | 0.16 (-0.14-0.64 | 0.25 | 0.16  (-0.14-0.64) | |
|  |  | OA Joint | 0 | 0 | 0 | 0 | 0 | 0 | 0 | 0 | 0.25 | 0.16 (-0.14-0.64 | 0.25 | 0.16  (-0.14-0.64) | |
| 14 | BO | Sham Joint | 0 | 0 | 0 | 0 | 0 | 0 | 0 | 0 | 0.38 | 0.18 (-0.06-0.81) | 0.38 | 0.18 (-0.06-0.81) | |
|  |  | OA Joint | 0.5 | 0.19  (0.05-0.95) | 0.13 | 0.13 (-0.17-0.42) | 0 | 0 | 0.13 | 0.13 (-0.17-0.42) | 1.13 | 0.23 (0.59-1.66) | 1.88 | 0.55 (0.58-3.17) | |
|  | Placebo | Sham Joint | 0.13 | 0.13 (-0.17-0.42) | 0 | 0 | 0 | 0 | 0.13 | 0.13 (-0.17-0.42) | 0.13 | 0.13 (-0.17-0.42) | 0.38 | 0.38 (-0.51-1.26) | |
|  |  | OA Joint | 0.63 | (-0.14-1.39) | 0.13 | 0.13 (-0.17-0.42) | 0 | 0 | 0.25 | 0.16 (-0.14-0.64 | 1.13 | 0.23 (0.59-1.66) | 2.13 | 0.64 (0.61-3.64) | |
| 70 | BO | Sham Joint | 0.25 | 0.16 (-0.14-0.64) | 0.13 | 0.13 (-0.17-0.42) | 0.25 | 0.16 (-0.14-0.64) | 0.13 | 0.13 (-0.17-0.42) | 1.00 | 0.19 (0.55-1.45) | 1.75 | 0.45 (0.68-2.82) | |
|  |  | OA Joint | 1.38 | 0.38 (0.49-2.26) | 0.38 | 0.26 (-0.25-1.00) | 0 | 0 | 0.63 | 0.26 (0.00-1.25) | 1.75 | 0.31 (1.01-2.49) | 4.13 | 1.03 (1.70-6.55) | |
|  | Placebo | Sham Joint | 0.38 | 0.18 (-0.06-0.81) | 0 | 0 | 0 | 0 | 0 | 0 | 0.50 | 0.27 (00.13-1.13) | 0.88 | 0.40 (-0.07-1.82) | |
|  |  | OA Joint | 1.88 | 0.30 (1.18-2.57) | 1.00 | 0.19 (0.55-1.45) | 0.88 | 0.23 (0.34-1.41) | 1.25 | 0.23 (0.59-1.66) | 2.38 | 0.32 (1.61-3.14) | 7.25 | 0.94 (5.03-9.47) | |

**Supplemental Table 2:** Radiographic scores (mean +/- SEM). These values represent raw data and differ from the values that were statistically analyzed therefore no data point correlates with a specific p-value.
